# Maiming by Firearms, etc.

**Published:** 1888-08-11

**Authors:** 


### Army Recollections.

*(Continued from p. 296.)*

### MAIMING BY FIRE-ARMS, Etc.

As it is generally assumed that this form of mutilation is accompanied by great pain and much local swelling and suffering, I am induced to say a few words on these points, and add generally that such is not always the case. A few illustrations will suffice for this purpose, and these are drawn rather from the observations of others than from any experience of my own. It is stated in Simpson's "Waterloo," that when Lord Anglesea saw struck at that battle he exclaimed, "I'm shot, by G—d!" Whereupon he was condoled with by his superior, the Iron Duke, with the interrogatory, "Are you, by G—d?" And most readers must be familiar with General Bounce's estimate of this situation. It is this: "Pain! the pain is nothing. A dislocated ankle is far more acute agony that it would take to kill an elephant—'tis but a touch to a trigger and the thing's done."

It appears, says the *Athenæum*, of June 8th, 1878, that the pain immediately produced by the passage of a bullet is usually slight. In some cases it is not felt at the entrance wound, but only at that of exit. A private of the 7th Fusiliers was in face of the enemy at Inkerman. A bullet pierced the lower and outer part of his neck, and tore its way out behind, between the upper angle of the scapula and the spine. An officer of the 2nd Battalion Rifle Brigade was behind him. No idea of having been shot entered the private's mind. He was not even aware of the wound he had received in front, but his sensations led him to suppose that the officer behind had pricked him with the point of his sword in the back. He turned round instantly to learn what this was done for, and was in time to see the officer in the act of falling. The bullet which had just passed through his own neck had struck the officer in the head and killed him.

The following incident occurred in the person of an officer with whom I was once associated. This officer was conversing at the time with Captain (now General) Wolseley and Captain Beresford in the trenches before Sebastopol, when a round shot carried off his arm. Captain Browne, the officer here referred to, jumped up from the ground and actually did not know of the loss he had experienced. He asked his comrades hurriedly, "What's the matter?" one of them replied, "Nothing."

A similar incident occurred to another officer at the Alma. He was shot through the arm, and never knew he was wounded till the battle was over; and Captain (now, I assume, Colonel) Montagu, tells us that "a soldier who was wounded in the cheek at one of the battles in Zululand, turned to his officer under the impression that he—the officer—had struck him with his sword on the cheek." If all one hears about the Knebel rifle be true, bullets from it will pass like a flash of lightning through the soft parts, and cause little or no pain. Or they may lead to fatal hæmorrhage, and as they smash completely, or rather, I believe, split up the longer bones —a most dangerous complication, as all military surgeons know—the shock and other complications must often inevitably lead to death.

Finally, as regards the attempt that was made on the life of the Duke of Edinburgh by one O'Farrell in Australia, he (the Duke), at once fell forward on his knees and hands, exclaiming, "Good God! I am shot, my back is broken." Speaking of the event afterwards the Prince said (the *Cruise of the Galatea*, pp. 407-14), that "when he heard the report of the pistol he did not feel that he was shot, but thought he had trodden on a Chinese cracker, and that when he fell forwards, the sensation was not that of falling, but as though he was being lifted up behind. It was only after he had raised himself by his hands into a sitting posture, and saw a man pointing a pistol at him, that he knew he had been shot, then, finding all sensation from the small of the back downwards had entirely gone and that there was an extraordinary coldness in his legs, which lay quite powerless, he concluded his back was broken. He never for a moment lost his consciousness, but was able to remark every circumstance that occurred; he felt no pain whatever, but suffered from a sense of suffocation, and when the bearers were taking him up to Government House, they had repeatedly to stop, to allow him to recover his breath." The pain of gunshot wounds being so inconsiderable, no determined or resolute malingerer will be deterred from using his firelock as a *dernier resort*.

